# Socio-economic determinants of household dietary diversity in a secondary African City: evidence from a community-based cross-sectional study in Bindura, Zimbabwe

**DOI:** 10.3389/fnut.2026.1808585

**Published:** 2026-09-30

**Authors:** Onismo Muzah, Maxwell Mudhara

**Affiliations:** African Centre for Food Security, School of Agricultural, Earth and Environmental Sciences, College of Agriculture, Engineering and Science, University of KwaZulu-Natal, Pietermaritzburg, South Africa

**Keywords:** Bindura, dietary diversity, food security, nutrition, urban household

## Abstract

**Background:**

The quality of diets in low-income African cities is shaped by more than food availability alone but also reflects household capability, the utility of infrastructure, consumption behaviour, coping mechanisms, and access to diverse sources of food. However, empirical evidence on the determinants of household dietary diversity in secondary African cities remains limited, despite the importance of understanding nutritional adequacy, which is vital for the growth and maintenance of bodily functions and for combating malnutrition, micronutrient deficiencies, and excessive energy intake.

**Objective:**

This study examined the socio-economic predictors of household-level dietary diversity in Bindura municipal area, Zimbabwe, using a secondary re-analysis of cross-sectional household survey data collected in October 2014 from 200 urban and peri-urban households.

**Methods:**

To achieve this objective, dietary diversity was measured using the FAO-developed dietary diversity survey based on a 24-hour recall period, and associations were estimated using descriptive statistics, chi-square tests, ANOVA, Spearman’s rank correlation, and ordered logistic regression.

**Results:**

Household dietary diversity was negatively correlated with household food insecurity, indicating that households experiencing greater food access constraints tended to consume less diverse diets. The ordered logit model results revealed that access to transport, water and electricity, sources of vegetables, number of meals per day, and household coping mechanisms were significant predictors of higher dietary diversity. In contrast, household asset value showed a weak positive association.

**Conclusion:**

These findings suggest that dietary diversity in Bindura is shaped primarily by food access conditions, basic infrastructure, and household responses to economic stress rather than by demographic characteristics alone. Therefore, household food and nutrition policy in secondary cities should be integrated with service delivery, household resilience support, and local food-system planning to improve diet quality among vulnerable urban populations.

## Introduction

Urban food and nutrition insecurity have become an increasingly important policy and research concern across sub-Saharan Africa, where rapid urbanisation is reshaping household livelihoods, food access pathways and diet quality. Between 2015 and 2021, the urban population grew by more than 397 million people, with over 90% of that growth occurring in developing regions (1, 2) and is projected to increase to 658 million by 2025 (3). The fastest growth is in secondary cities. Zimbabwe has also experienced considerable urbanisation, with the 2022 national census data indicating that the proportion of the urban population had increased to 38.6% in 2022 from 33% in 2012 (4, 5). While urban households are often assumed to have better access to markets compared to rural populations, low-income households in many African towns and cities continue to face severe constraints in accessing safe, nutritious, and diverse diets (6, 7). This includes increased consumption of low-cost starchy staples and vegetables, as well as energy-dense, highly refined foods high in fat and sugar, and low in micronutrients (8–10), with limited consumption of nutrient-dense foods such as animal-source products, fruits and dairy products. These consumption challenges also affect peri-urban households, which heavily rely on the urban ecosystem for opportunities and services.

Globally, the importance of dietary diversity in addressing malnutrition and promoting overall health is increasingly being recognised (11, 12). The World Health Organisation (WHO) reports that in 2022, an estimated 2.5 billion adults worldwide were overweight, with 890 million categorised as obese, whereas 390 million were underweight (13). This reflects the extent of global malnutrition. UNICEF (14) Zimbabwe data indicates that in 2024, about 26.7% of children under the age of five were stunted, and the prevalence of child wasting increased from 4.1% in 2023 to 4.9% in 2024 in rural areas and from 3 to 5.6% in urban areas, highlighting a deteriorating nutrition situation. Overweight and obesity are increasing at a rapid rate in Zimbabwe and presenting a significant public health risk alongside other types of malnutrition, including underweight and macronutrient deficiencies (14), in what has been termed the triple burden of malnutrition.

Poor dietary quality in urban areas is thus not simply *an aspect* of food availability, but a consequence of *quality*, affordability, low-income threshold and the structure of the urban food system. In Zimbabwe, there is a range of intertwined socio-economic, political, spatial and environmental factors that explain this, including the economic downturn, chronic poverty and food price inflation, inequities in the food system value chain, climate shocks on food production and prices, and other factors (15). The lack of dietary diversity is a chronic problem among low-income populations in developing countries, as their diets are primarily composed of starchy staples and include little to no protein-rich foods, as well as few fresh fruits and vegetables (16, 17), due to affordability constraints. The recommended healthy diets contain a variety of foods that provide nutrients and bioactive compounds essential for health, satisfactory in dietary energy sources such as proteins, carbohydrates, and fats, and they are moderate in nutritional elements that are harmful to health if consumed in excess (18–20). However, the lack of purchasing power is one of the key factors hindering the urban population in Sub-Saharan countries from obtaining the diverse foods needed for an adequate caloric intake compared to rural constituencies, who often grow and process their own food. The criticality of dietary diversity is recognised within the Sustainable Development Goals (SDGs), specifically SDG 2 (Zero Hunger) and SDG 3 (Good Health and Well-being), which emphasises improving nutrition and achieving food security for all by 2030. However, Zimbabwe is lagging and not on track to achieve these ambitious targets for both the rural and urban populations by 2030.

Households are food secure when they have access throughout the year to adequate quantities and a variety of safe foods that their members require for active and healthy lives (21, 22). In 2023, an estimated 2.33 billion people globally faced moderate or severe food insecurity, and among those, over 864 million experienced severe food insecurity, to the extent of going without food for an entire day or more at a time (23). On a household level, this suggests a high degree of vulnerability to food insecurity. Zimbabwe continues to face high levels of food and nutrition insecurity among its vulnerable population, specifically children, older people, women, and people living with disability (24). An estimated 2.4 million people in Zimbabwean towns and cities were struggling to meet their basic food needs, coping by skipping meals, selling their hard-earned assets and taking their children out of school (25). These challenges are intensified by broader political-economic stresses, including chronic poverty, inflationary burden, unstable employment, poor agricultural productivity due to climate change and variability, and periodic disruptions in food markets. Poverty remains one of the leading primary causes of vulnerability to food and nutrition insecurity in the country, as well as unreliable and precarious livelihoods (98). This is a paradigm that recognises and balances food supply, demand, entitlement, access and consumption (15). Hence, its relevance and applicability to urban food and nutrition insecurity.

Historically, food security in Zimbabwe has often been equated with achieving self-sufficiency in staple maize production, stability in agricultural input prices, adequate rainfall per season, and income endowment to meet food basket prices for urban households. This is no longer the case as the urban population in Zimbabwe continues to face increasing consumption challenges driven by economic shocks, high unemployment, and rising costs of living, which disproportionately impact women-headed households, young people, and individuals with disabilities. (26). The most affected often rely on wage labour, informal trading, cross-border and overseas remittances, small-scale production and precarious income-generating activities to meet their food needs. Under such conditions, the ability to secure a diverse diet depends on far more than household income alone, (27) but shaped by socio-economic, cultural, and environmental variables. This includes education, household assets, coping strategies, occupation, transport and infrastructure, and market value chains. However, the interaction of these factors remains insufficiently understood in secondary cities, particularly outside the country’s largest towns and cities.

Household Dietary diversity (HDD) is an essential aspect of the broader concept of food security, defined by a variety of foods within and across food groups consumed in a particular period (28), to ensure adequate intake of essential nutrients that can promote good physical health and mental development. It is positively linked with the pillars of food security, such as availability, access and utilisation (22, 29, 30). This is because food security and nutrition are closely interwoven, and one crucial element that clarifies this link is the quality of the foods people eat (31).

However, there is no consensus on the optimal standardised measure for dietary (32, 33), but dietary diversity is a qualitative measure of food consumption that reflects the variety of foods a household has access to and serves as a proxy for the nutritional adequacy of an individual’s diet (28). The aggregate of the various food items consumed by an individual or household forms a composite index, known as the Household Dietary Diversity Score (HDDS). The HDDS aggregates food groups, including cereals, roots and tubers, vegetables, fruits, meat, eggs, legumes, milk and milk products, oils, fats, and sugars, consumed by any household member within a 24-h to 7-day period [(34); Swindale]. The score is an essential indicator of an individual’s or a household’s food security.

The standard measures of food insecurity include the Household Food Insecurity Access Scale (HFIAS) (35), and the Food Insecurity Experience Scale (FIES) (36) which captures economic food access and food sufficiency, the Household Dietary Diversity Score, which captures the quality of diets (37), and the months of the Adequate Household Food Provisioning Indicator (38) a proxy measure of household food access, and the Coping Strategy Index (CSI) captures adaptive mechanisms devised by households when they face food shortages (39). These metrics are interrelated and capture different combinations of food security dimensions, but are not equivalent (40), but are all widely recognised as micronutrient adequacy proxies influenced by a host of structural and household-level aspects (28). Comparative food and nutrition security studies by (41–44) have used these metrics in Zimbabwe. These indices are relevant to Zimbabwe’s nutrition policy environment because they capture the household-level dimensions entrenched in national food and nutrition strategies. Zimbabwe has developed and updated its National Nutrition Strategy (2014–2018) and (2023–2025), which integrates different sectors and reflects its systems approach to food security, providing a direct policy basis for these measures. The strategy provides a multi-sectoral guiding framework for coordinated food and nutrition security interventions in Zimbabwe (45). Other key policies, such as the Food and Nutrition Security Policy 2012, the National Agriculture Policy Framework 2019, and the 2018 National Development Strategy, which underpins Vision 2030, help translate the broader objectives of the National Nutrition Strategy into measurable household-level outcomes.

Existing studies in Uganda, (12) Ethiopia (46), Nigeria (47) have documented associations between dietary diversity and income, education, market access, household composition, asset ownership and livelihood dynamics. However, much of the evidence is drawn either from rural areas or large urban cities, leaving secondary cities comparatively neglected. Thus, the Bindura Municipal area in Zimbabwe lacks empirical evidence on the distribution of dietary diversity and food security among urban and peri-urban households. This matters because secondary cities occupy a distinctive position between rural and large city systems, often combining features of both: agrarian linkages, peri-urban food production, emerging markets, uneven infrastructure, and high livelihood vulnerability. These hybrid characteristics can create food access dynamics distinct from both large cities and rural villages. Therefore, the policy relevance of secondary cities is substantial, yet empirical evidence on household dietary diversity in such contexts remains limited. Moreover, we argue that, although urban and peri-urban areas are conceptually distinct, the nexus between them is more insightful to analyse, especially when investigating micro-level dietetic practices among low-income households. This structural duality raises important empirical questions: which socio-economic factors most strongly shape dietary diversity in such contexts, and through which mechanisms do they operate?

Bindura, a secondary urban city, provided useful context for examining these questions. Despite a substantial body of literature on food security and dietary diversity in rural areas, far less attention has been given to household consumption challenges in secondary cities, despite their rapid growth and strategic role in national food systems. Prior studies in Zimbabwe have exclusively focused on rural or urban food security (4, 44, 48, 49) but very few (13, 50) has focused on urban household dietary diversity. Secondary cities surrounded by scattered peri-urban areas occupy a distinctive position between rural environments and large cities and often combine agrarian livelihoods with emerging urban markets, creating hybrid food access and availability in which households rely on both their own production and market access. Understanding the determinants of dietary diversity in such a context is important for two reasons. First, it helps identify the socio-economic mechanisms through which urban households gain or lose access to more diversified diets. Second, it contributes to knowledge of urban food systems by showing how household-level nutritional outcomes are shaped within specific urban food systems rather than solely by income.

Against this background, the objective of this study is to examine the socio-economic determinants of household dietary diversity in Bindura, Zimbabwe, using household survey data from urban and peri-urban locations. Specifically, the study investigates how human capital, livelihood structure, household assets, expenditure patterns and food sourcing pathways are associated with household dietary diversity. The study contributes new empirical evidence to an under-researched segment of African urban food systems by focusing on a secondary city and offers policy-relevant insights for improving food and nutrition security in Zimbabwe urban settings.

## Methods

### Study setting, design and participants

The study was conducted in the Bindura Municipality, a secondary city in Zimbabwe that includes both urban and peri-urban settlements and exhibits a mixed livelihood structure combining wage employment, small-scale farming, and informal economic activities. The Municipality has an estimated population of 331,667 for the year 2022, compared to the 2012 Census population of 168,894 people, and an estimated annual growth rate of 2.3% (51). The analysis presented in this paper is based on a secondary re-analysis of household survey data collected in October 2014 as part of the corresponding author’s earlier thesis research. It is therefore interpreted as a historically premised analysis of structural predictors of dietary diversity in Bindura rather than a current estimate of the household food and nutrition situation in 2026. The area is essential given the social, economic, demographic, and cultural context that influences dietary diversity outcomes. Bindura is both a mining and an agricultural town, where maize is the main staple crop. Individuals in the Bindura Municipal area earn a living from various sources, including mining companies, artisanal mining, processing companies, non-governmental organizations (NGOs), casual labour, buying and selling, private and public sector employment, roadside survivalist informal trading, the services sector, informal microenterprises, and agriculture.

The study employed a cross-sectional quantitative design to collect primary data from 200 households with knowledge and understanding of household consumption and socioeconomic position. To ensure a representative sample, a multi-stage sampling procedure was used, stratifying residential areas within the municipality by location. Thus, in the first stage, four urban and two peri-urban areas, namely Chipadze, Chiwaridzo, Aerodrome, Shashi Ville, Gulliver, and Nyamakura, were identified. The second stage was to identify household heads responsible for food preparation or household expenditure decisions. In the final stage, at least 33 households were randomly selected from each urban location and 34 from each peri-urban location, for a total of 132 urban and 68 peri-urban respondents. This approach captured diverse socio-economic conditions across the municipal area, including urban and peri-urban areas. The study employed the (52) quantitative formula for selecting the study sample size with a confidence precision interval level of 95%, as illustrated in Equation 1 below:

$$\;\;n=\frac{N}{1+N{\left(e\right)}^{2}}\;\;\;\;$$ (1)

Where N is the total sample, 𝑛 signifies the total number of households, and 𝑒 denotes the error (0.05% error). The 𝑒 was set to 0.05 because the variation among households was unknown. Therefore, for a sample of 200 households, the minimum sample size for reliable interference is 133. The Yamane sample dimension formula is used to regulate the minimum sample required for a study when the size of the population is identified, and a desired level of precision is estimated (53).

The data collection was conducted in October, corresponding with a lean season in Zimbabwe, during which households face constraints in accessing food (54). Data was collected using a semi-structured questionnaire with both open and closed questions, administered through face-to-face interviews. The questionnaire was adapted from the United Nations’ Food and Agriculture Organisation (55) household dietary diversity measurement and related household socioeconomic and demographic characteristics developed by the authors with reference to (56, 57), and (58). To accurately capture household consumption behaviour, the respondent was assisted by a household member responsible for food purchases, meal preparation, and meal processing, under a defined protocol.

### Measurement of household dietary diversity score

A 24-h data recall HDD was measured using the Household Dietary Diversity Score, which reflects a household’s economic ability to access a variety of foods (55), based on the WHO food group categorisation (59, 60). A scale of 12 food groups which includes (i) cereals, (ii) root crops, (iii) tubers, (iv) vegetables, (v) fruits, (vi) meat, (vii) eggs, (viii) fish, (ix) legumes, (x) milk and products, (xi) fats and (xii) sugar was used in assessing the dietary diversity of the respondents, and the dietary diversity scores for the respondents were estimated using (28), where a single point is awarded to each of the food groups consumed over the reference period. The number of groups reported was then summed up to obtain an HDDS (0–12) for the whole household, where (22, 55) LDD was defined as DDS ≤ 3, MDD as DSS between 4 and 6, and HDD as DSS ≥ 7, indicating food security. Consuming at least four food groups increases the likelihood that foods from animal sources, fruits, and vegetables were consumed in addition to staple foods (28, 47). At the same time, household economic food access and sufficiency were assessed separately using the 9-item Household Food Insecurity Access Scale (HFIAS), which reflects household food security for the previous month (33). The HDDS and HFIAS, thus, estimated related but distinct dimensions of household food and nutrition security. The HDDS *captured* dietary diversity and quality, whereas the HFIAS *evaluated* food access insecurity.

Table 1 below presents the 12 categories of food groups.

**Table 1:** Household dietary diversity food groups.

Food groups
1. Any sadza, bread, rice, noodles, biscuits, or any other foods made from millet, sorghum, maize, wheat, or any other locally available grain
2. Any potatoes, yams, manioc, cassava, or any other foods made from roots
3. Any tubers
4. Any vegetables
5. Any fruits
6. Any beef, pork, lamb, goat, rabbit, wild game, chicken, duck, or other birds, liver, kidney, heart, or other organ meats
7. Any eggs
8. Any fresh, dried fish or shellfish
9. Any foods made from beans, peas, lentils, or nuts
10. Any cheese, yoghurt, milk, or other milk products
11. Any food made with oil, fat, or butter
12. Any sugar, coffee, tea, or honey

Source: adapted from (28). If the answer is ‘YES’, 1 point is awarded; if the answer is ‘NO’, a nil point is awarded.

### Empirical model specification

The ordered logit regression model was a better fit and appropriate in analysing the household consumption and nutritional outcomes in Zimbabwe because of the nature of the dependent variable, following similar studies conducted by Sambo et al. (61) and Jateno et al. (62). The dietary diversity score was computed as a dependent variable, and three dietary diversity categories were created: explicitly low, medium and high dietary diversity using the methodology defined by Swindale and Bilinsky (37). The cumulative logistic regression model, which is well known as the proportional odds model (POM), is the most widely used type of ordinal logistic model (63). In this study, the POM was used to estimate the cumulative probability that a household is in each dietary diversity category or below, given a set of covariates. Adoption of this categorisation of the HDDS in this study is further justified by the fact that *several* studies conducted in Sub-Saharan Africa by Ambaye et al. (64), Balana et al. (65), Lelisho et al. (66), Schwei et al. (67), Taruvinga et al. (68), have also adopted the same categorisation of the HDDS. The ordered logit model estimates the underlying tendency of an observed phenomenon, considering a vector of explanatory variables and a random error term (62, 69). The ordered logistic regression model was fitted to the data to identify factors significantly associated with household dietary diversity. The equation of the ordered logit model regression is specified as.

$${Y_{i}}^{*}=\beta_{0}+\sum_{k=1^{K}}\;\beta_{k}X_{\mathrm{ki}}+\varepsilon_{i}$$ (2)

Where Y* is unobserved, *X_k_* is a vector of independent variables, *β* denotes coefficients to be estimated, and *ε* denotes a random error term. There is a random disturbance term that, in this case, follows a standard logistic distribution. This reflects the fact that relevant variables may be omitted from the equation or not perfectly measured. The Ordered Logit Model estimates part of the above:

$${Y_{i}}^{*}=\beta_{0}+\sum_{k=1^{K}}\;\beta_{k}X_{\mathrm{ki}}$$ (3)

From the above model, the observed or defined categorical variable Yi is determined as follows:

*y =* 1 if y* *≤ μ*1.

*y* = 2 if *μ*1 < y* ≤ *μ*2.

*y* = 3 if *μ*2 < y* ≤ *μ*3.

*y* = *j* if *μj*−1 < y*.

In case *y* is observed in j number of ordered categories, *μs* are unknown threshold parameters differentiating the.

adjacent categories to be assessed with βs; then μ1, μ2 and μ3 represent the different levels of the HDDS that are to be estimated (70). The general form for the probability that the observed y falls into category j and the μs and the βs are to be estimated with an ordered logit model is.

$$\mathrm{Pr}(Y_{i}=j)=\Lambda (\mu_{j}-\sum_{k=1^{K}}\;\beta_{k}X_{\mathrm{ki}})-\Lambda (\mu_{j-1}-\sum_{k=1^{K}}\;\beta_{k}X_{\mathrm{ki}})$$ (4)

Where:

- Pr(Yᵢ = j) is the probability that household *i* falls in dietary diversity category *j*,
- Λ(.) is the logistic cumulative distribution function,
- μ_j_ and μ_j−1_ are threshold parameters,
- β_k_ are parameters to be estimated,
- X_ki_ are the household *i* explanatory variables,

### Description of variables

The variables used in the ordinal logit model are listed in Table 2. The dependent variable for the model is the HDDS categories (37) low dietary diversity category (≤3 food groups), medium diversity category (4–5 food groups), and high diversity category (≥6 food groups), measured by the number of food groups consumed by household members in the 24 h preceding the interview.

**Table 2:** Explanatory variables used in the regression model.

Variable name	Variable description	Measurement	Expected signs	
			LDD	HDD
Age	Household coping strategy with income deficits	Years	+/−	+/−
Location	Location of the household	1 = Peri-Urban; 2 = Urban	−	+
HH size in adult equivalents	Household size	Number of members	+/−	+/−
Educational level	Household head education	Number of years in school	−	+
Occupation	Household head employment status	0 = unemployed; 1 = Employed	−	+
Transport	If the household pays for Transport	0 = NO; 1 = YES	+	−
Water and Electricity	If the household has access to water and electricity	0 = NO; 1 = YES	−	+
Household income	Households’ monthly income	Amount in USD/ZAR 0 ≤ 100; 1 = 101–300; 2 = 301–500; 3 ≥ 500	−	+
Monthly Spending on Food	Household expenditure on food	Cost of expenditure in USD/ZAR	−	+
Maize meal source	Household source of supply for maize meal	1 = Own production; 2 = Formal and informal markets,	+	+
Vegetable sources	Household source of supply for vegetables	1 = Own production; 2 = Formal and informal markets,	+	−
Total value of assets	Total value of physical household assets	Value in USD/ZAR	−	+
Number of meals	Number of meals per day in normal circumstances	1 = One meal; 2 = Two meals, 3 = Three meals	−	+
Household income coping strategy	Household coping strategy with income deficits	0 = sell livestock; 1 = sell assets; 2 = use household cash savings; 3 = Borrow money from friends and family; 4 = reduce spending	+/−	+/−

LDD, low dietary diversity; HDD, high dietary diversity, respectively.

## Results and discussion

### Descriptive statistics of household nutritional security

Figure 1 below presents a radar summary showing the proportions of households consuming different food groups in the study area, based on a 24-h dietary recall.

**Figure 1:**
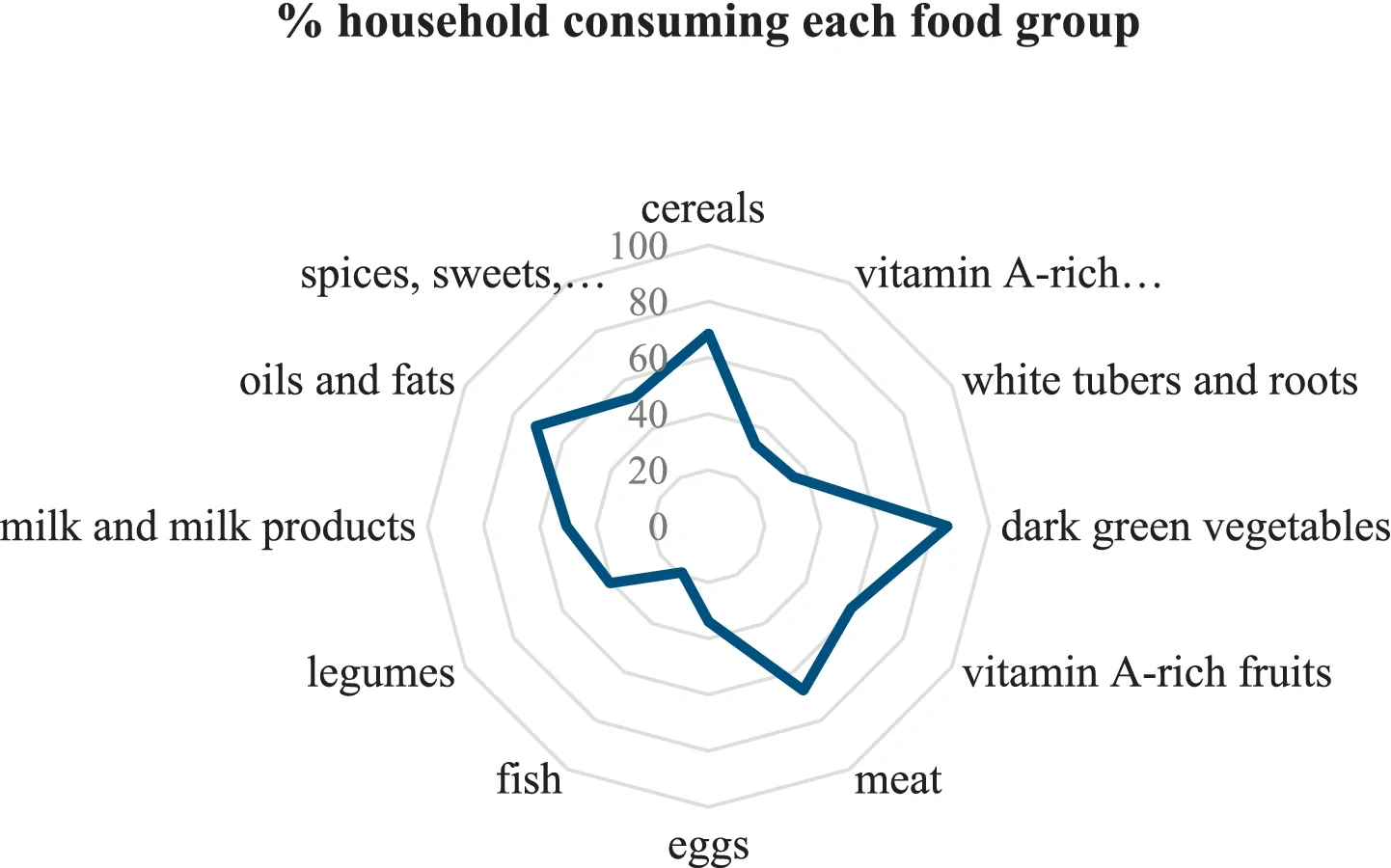
Percentages of households that consumed different food items.

The observed distribution indicates that the consumption of dark green leafy vegetables (85%), oils and fats (71%), cereals (68.5%), and meat (67.5%) was relatively high. Modest consumption was observed for vitamin A-rich fruits (58.5%), spices, sweets and beverages (53%), and milk and milk products (50.5%). This is relative to lower proportions of households that consumed legumes (40.5%), white roots and tubers (35%), eggs (34%), vitamin A-rich vegetables (33.5%), and fish (19%). The observed distribution suggests that, on average, urban households’ diets are mainly rich in dark green leafy vegetables, fats, staple cereals, and meat, at the expense of highly required milk, eggs, fish, vitamin A vegetables, and fruits. This may imply that a lack of starchy staples and carbohydrates mainly characterises low dietary diversity among urban poor households. Most households surveyed were food insecure, with 26.5% having low dietary diversity, 57% having medium diversity, and only 16.5% having a high nutritional diversity score, as shown in Table 3.

**Table 3:** Dietary diversity frequencies found in the study.

Measure	Low DD	Medium DD	High DD
Dietary diversity score	0–4	5–9	10–12
Percentage of respondents	26.5	57%	16.5

*n* = 200. Data source: Household Survey (2014).

These results indicate that HDD is moderate but imbalanced, as the diets are heavily dependent on staple cereals and vegetable-crop-based foods. On the other hand, the recommended animal-source and nutrient-laden foods, such as fish, eggs, milk, and milk products, are consumed less frequently. The higher proportion of cereal and vegetable consumption indicates poverty and a reliance on affordable, readily available foods to meet basic nutritional needs, without providing any nutritional benefits. Similarly, the low intake of protein and micronutrient-rich foods suggests affordability constraints, incomplete dietary quality, and nutritional vulnerability. The high and low diversity scores recorded indicate that most households experience a deficiency in dietary diversity, highlighting fundamental financial, market, and access barriers distinctive of low-income towns such as Bindura. The results indicate that dietary diversity is low, thereby increasing the risk of food insecurity in the area. Among the various food groups, including starches, vegetables, fruits, animal-sourced foods, fats, and legumes, the study reveals that the interviewed households could afford mostly starch- and carbohydrate-rich foods because they are readily available and cheaper on the market. This result is consistent with a study by (71), in Harare, which revealed that household dietary diversity was very low, with an estimated 68% of households consuming fewer than 12 different foods in the previous 24 h.

### Association between household socio-economic characteristics and dietary diversity

The Pearson chi-square results (Table 4) reveal several statistically significant relationships and correlations between household dietary diversity and key socio-economic variables, specifically employment status, source of vegetables, number of daily meals, food storage, and residential location. These findings provide strong evidence that household-level socio-economic dynamics and the means of food access substantially shape dietary diversity outcomes among urban and peri-urban households in Bindura Municipality. The occupation status of the household head was correlated significantly with dietary diversity (*p* < 0.05), highlighting the critical role of employment in determining food access and nutritional adequacy at the micro-level. Households with employed heads recorded higher dietary diversity scores than those with unemployed heads, indicating greater purchasing power, regular income streams, and improved access to a broader range of food groups. In the same vein, the source of vegetables for urban and peri-urban households also showed a statistically significant relationship with dietary diversity (*p* < 0.10), particularly in periods of market and food price volatility, which is often the case in Zimbabwe. The number of meals consumed per day was highly significant (*p* < 0.01) and positively correlated with higher dietary diversity. Households enjoying three meals a day exhibited improved DD, equated to those having one or two daily meals. This relationship likely reflects broader food access capabilities and intra-household food security dynamics.

**Table 4:** Dietary diversity score and household nutritional security parameters.

Variable	Categorical	Dietary diversity			*n*	*X* ^2^
		Low %	Medium %	High %		
Gender	Female	22.4	63.2	14.5	76	ns
	Males	29.0	53.2	17.7	124	
Marital status	Single	26.5	59.2	14.3	49	ns
	Married	26.5	56.3	17.2	151	
Occupation	Unemployed	65.6	28.1	6.3	32	**
	Employed	19.0	62.5	18.5	168	
Maize source	Own production	30.2	57.3	12.5	96	ns
	Multiple sources	23.1	56.7	20.2	104	
Vegetable source	Own production	19.8	57.4	22.8	101	*
	Multiple sources	33.3	56.6	10.6	99	
Coping strategy	Sell assets	4.5	5.0	1.5	22	ns
	Other strategies	22.0	52.0	15.0	178	
Number of meals a day	Three meals	12.0	63.2	24.8	125	***
	Two meals	50.0	47.3	2.7	74	
	One meal	100	0	0	1	
Education	Non formal	36.4	36.4	27.3	11	ns
	Formal	25.9	58.2	15.9	189	
Food stored	No	36.9	52.4	10.7	103	***
	Yes	15.5	61.9	22.7	97	
Location	Peri-urban	54.4	39.7	5.9	68	***
	Urban	12.1	65.9	22.0	132	

***, ** and * indicate statistical significance at the 1%, 5% and 10% levels, respectively. ns = not statistically significant. Data source: Household Survey (2014).

The capacity to store food was another strong determinant (*p* < 0.01) of dietary diversity. Households that stored food were more likely to record higher dietary diversity scores than those that did not. The residential location demonstrated a statistically significant relationship with dietary diversity (*p* < 0.01), with urban households in the study recording a higher dietary diversity than those in peri-urban areas, highlighting spatial inequalities in food access.

### Extent of household nutritional security: ANOVA test results

The ANOVA results presented in Table 5 specify further insights into the extent to which key socio-economic characteristics influence household nutritional security, as proxied by dietary diversity levels. Statistically significant differences were observed across household cohort groups in the age of household headship, monthly expenditures on food, monthly expenditures on non-food items, and the total value of household asset endowment. These variables collectively highlight the economic and demographic gradients driving inequalities and disparities in dietary quality among urban and peri-urban households in Bindura Municipality. A statistically significant difference was also observed across age groups (*p* < 0.10), suggesting that household age plays a facilitating role in shaping dietary diversity. Monthly food spending was strongly associated with household nutritional security (*p* < 0.01). Every month, households in the high dietary diversity category spent significantly more on food (US$120.33) compared to those in the medium (US$99.56) and low (US$49.36) cohorts.

**Table 5:** ANOVA test results for the extent of household nutritional security.

Variables	Low	Medium	High	Significant level
*n*	53	114	33	(Anova)
		Mean		
Age (years)	38.92	42.11	38.09	*
Household size in adult equivalence	4.4	4.6	4.6	ns
Land size	1.28	1.78	2.03	ns
Monthly spending on food (US$)	49.36	99.56	120.33	***
Total household income (US$)	1,200.98	1,048.38	932.42	ns
Household expenditure on nonfood items (US$)	3.34	5.82	6.64	***
Value of households Assets (US$)	3,018.08	3,788.07	5,483.88	***
Hectares allocated to food crops	0.74	1.15	1.00	ns
Hectares allocated to cash crops	0.37	0.77	0.69	ns

*** and * mean significant at 1 and 10% levels of significance, respectively. Ns not statistically significant. Data source: Household Survey (2014).

Expenditure on non-food items was also statistically significant (*p* < 0.01) and increased with dietary diversity. This variable feasibly captures broader household welfare and purchasing power beyond direct food access alone. Furthermore, household asset value showed a similarly strong positive association with nutritional security (*p* < 0.01). Thus, households with higher dietary diversity had substantially higher asset values, averaging US$5,483, compared to US$3,018 in low-diversity households (Table 6).

**Table 6:** Relationship between household dietary diversity and household food security.

Variable pair	Coefficient	*p*-value	95% CI
Spearman’s rho			
Dietary diversity score vs. food insecurity score	−0.310	***	−0.433 to −0.175
Pearson correlation			
Dietary diversity score vs. food insecurity score	−0.317	***	−0.436 to −0.186

Spearman’s rho was used as the primary measure of association because of the ordered nature of the variables and the sparsity observed in the dietary diversity HFIAS contingency table. Negative coefficients indicate that higher food insecurity is associated with lower household dietary diversity. *** mean significant at 1% levels of significance, respectively. Ns not statistically significant. Source: Household Survey (2014).

### The nexus between dietary diversity and food security

To examine the nexus between household dietary diversity and household food security, a non-parametric correlation analysis was conducted using Spearman’s rank correlation coefficient. This method was preferred because both variables are ordinal. The analysis revealed a statistically significant negative correlation between the household dietary diversity score and the household food insecurity score (*ρ* = −0.310, *p* < 0.01). The 95% confidence interval ranged from −0.433 to −0.175, indicating a consistent inverse association. This suggests that households experiencing increased consumption difficulties tend to have lower dietary diversity scores, while those with lower consumption challenges are more likely to have higher dietary diversity. The Pearson correlation produced a similar result (*r* = −0.317, *p* < 0.01), highlighting the robustness of the observed negative relationship. This implies that household food access problems are closely linked to poorer dietary diversity outcomes in the Bindura municipal area.

### Determinants of household dietary diversity: ordered logistic regression model results

The ordered logistic model was estimated to determine the socio-economic characteristics and resource endowments that influence household dietary diversity, as presented in Table 7.

**Table 7:** Determinants of household nutrition dietary diversity using the ordered logistic regression model.

Variable	Coef	Wald	Std. err	*p* > *z*	Odds ratio	95% CIa
Age	0.0022	0.0173	0.13	0.898	1.0022	0.9689–1.0367
HH size in adult equivalents	0.0095	0.1061	0.09	0.929	1.0096	0.8200–1.2428
Educational level	−0.0042	0.0542	−0.08	0.939	0.9958	0.8954–1.1074
Total value of assets	0.0715	0.0042	1.69	0.091*	1.0001	1.0000–1.0002
Spending on food	0.0012	0.0031	0.38	0.701	1.0012	0.9952–1.0072
Location						
Urban	0.5254	0.4629	1.14	0.256	1.6910	0.6826–4.1905
Occupation						
Employed	0.2243	0.5514	0.41	0.684	1.2514	0.4247–3.6876
Transport						
Yes	1.3287	0.4367	3.04	0.002**	3.7768	1.6045–8.8864
Water and electricity						
Yes	1.1678	0.5364	2.18	0.029**	3.2148	1.1236–9.1987
Household income						
101–500	0.6430	0.5084	1.26	0.206	1.9022	0.7023–5.5275
>500	0.5720	0.6684	0.86	0.392	1.7718	0.4787–6.5564
Maize meal source						
Market/other sources	0.3456	0.3706	0.93	0.351	1.4128	0.6832–2.9238
Vegetable meal source						
Formal and informal markets	−1.3199	0.3737	−3.53	0.000***	0.2672	0.1284–0.5576
Number of meals						
Three meals	1.3807	0.4077	3.39	0.001***	4.7702	1.7891–8.8449
Household income coping strategy						
Asset/stress coping	−0.7866	0.3761	−2.09	0.036**	0.4554	0.2179–0.9517

*n* = 200 LR chi^2^(15) = 125.12, Pseudo *R*^2^ = 0.33, Log likelihood = −129.47, Prob > chi^2^ = 0.0000. *, **,*** means the coefficient is statistically significant at 10, 5, and 1% levels, respectively. Data source: Household Survey (2014).

Relevant procedures for model suitability tests were conducted to assess the model’s fit to the data. The likelihood ratio test, based on the −2 Log Likelihood, was used to assess the model’s goodness of fit. The model had a log-likelihood of −129.47, which was significant, as reflected by the significant Chi-square value (*p* < 0.01), suggesting that all estimated exogenous variables jointly explained the dietary diversity status of urban and peri-urban households (Table 7). The pseudo-R-squared is approximately 33%, which is relatively low for cross-sectional data. Multicollinearity was not a problem, as the variance inflation factors (VIFs) of all independent variables ranged from 1.22 to 2.22 and averaged 1.57, confirming that the model did not suffer from multicollinearity. The proportional odds assumption was estimated using the Brant test, which provides an assessment equivalent to the test of parallel lines in ordinal regression. The overall Brant test was not statistically significant (*χ*^2^ = 12.68, df = 12, *p* = 0.393), indicating that the parallel regression assumption was not violated.

### Robustness tests

To assess the robustness of the ordered logit result, several additional checks were conducted. First, the baseline model was re-estimated using an ordered probit model to test whether the direction and significance of the explanatory variables were sensitive to the choice of ordinal response model. The coefficient of *SID* is the same in both sign and significance as in the original baseline regression, meaning that the positive impact of the predictor variables on dietary diversity is relatively reliable. The results were substantively similar, with transport access, access to water and electricity, vegetable source, and number of meals continuing to have a significant positive effect on dietary diversity. At the same time, total household asset value remained positive at the 10% significance level. This consistency suggests that the choice of the ordinal response model does not drive the main findings. The ordered probit model was collectively significant [Wald *χ*^2^ (14) = 112.44, *p* < 0.01], indicating that the explanatory variables retained substantial predictive value under an alternative link function. These results strengthen confidence in the substantive conclusion that household dietary diversity in Bindura is primarily shaped by access conditions, infrastructure, and food-sourcing pathways rather than by demographic characteristics alone.

### Model odds ratios

The regression results exhibited that asset endowment, access to transport, water and electricity, source of vegetables, number of meals per day, and household coping strategy were significant drivers of household dietary diversity. Although not significant at the 5% level, the total value of household assets was positively associated with dietary diversity at the 10% level. Substantively, the positive sign suggests that households with greater asset endowments were more likely to attain higher dietary diversity than those without valuable, productive assets. Households with access to transport had 3.78 times higher odds of being in a higher dietary diversity category than households without transport access.

Similarly, households with access to water and electricity had 3.21 times higher odds of belonging to a higher dietary diversity category compared with households without these services. On the other hand, households whose vegetables were obtained mainly from formal and informal markets had 73.3% lower odds of being in a higher dietary diversity category relative to households whose vegetable supply came from their own production. Households reporting three meals per day had 4.77 times higher odds of falling into a higher dietary diversity category than households in the reference meal category. Lastly, households using asset- and stress-coping strategies had 54.5% lower odds of being in a higher dietary diversity category than those using financial coping strategies, such as savings.

## Discussion

The study examined the socio-economic and resource-related determinants of household dietary diversity in a secondary urban context using literature, descriptive statistics and an ordinal logit regression model. The empirical research in this paper is carried out in three main parts: Firstly, the ordinal logit regression method is used to estimate the drivers of household dietary diversity outcomes in the urban context. Secondly, the proportional odds assumption was estimated using the Brant test, which provides an assessment equivalent to the test of parallel lines in ordinal regression. Thirdly, we use a robustness check of the ordered logit results to test whether the direction and significance of the explanatory variables are sensitive to the choice of the ordinal response model.

### Factors influencing dietary diversity in urban and peri-urban households

The research findings highlight the significance of asset ownership for urban households in consumption and nutritional outcomes. Households with greater asset endowments are better positioned to smooth consumption, access a variety of food, and endure market disruptions through their capacity to generate revenue. This is consistent with studies of Anyanwu et al. (72) in Ethiopia, Ramafoko et al. (73) in South Africa and Kiboi et al. (74) in Kenya. They observed that household productive assets that generate regular income serve as a proxy for wealth, provide access to decision-making, and are a key strategy for improving diet quality. This nexus is expected, as asset ownership serves as productive resources, purchasing power in food markets, and as a cushion against income and consumption shocks. An increase in asset ownership strengthens households’ purchasing power, enabling them to spend more on food (75). However, Murendo et al. (76) in their study in Zimbabwe, offered a different perspective, arguing that during consumption shocks, assets invariably increase household dietary diversity only among the poorest households. In the context of urban food poverty and attrition, assets can be converted into cash, thereby reducing dependence on short-term coping strategies that often degrade diet quality. This further validates evidence from (77, 78) that both productive and non-productive asset endowments enhance household food and nutrition security, as well as improved liquidity and risk-absorption functions. Therefore, to improve urban household dietary diversity, it may be essential to substantially accumulate both physical and financial assets, such as equipment, machinery, vehicles, and stocks and savings.

In many urban areas in the Global South, people rely on various modes of transport to access goods and services; hence, in this study, access to transport was a driver of household dietary diversity. This suggests that transport improves physical access to markets and food outlets, thereby increasing the likelihood that households can obtain a more diverse diet. A household’s mode of transport for acquiring food is related to the local food environment and the quality of the diet (79). In a similar vein, (79) cited in (80) found that households with better access to transport facilities, such as private vehicles, were more likely to have higher dietary diversity than households that rely on public transport, walking, or biking. This suggests that mobility matters for food and nutrition security in urban environments. Transport improves access to formal and informal food markets, reduces transaction costs, widens the range of foods available to households, and enables more flexible food purchasing behaviour. In urban and peri-urban environments, where households rely heavily on food markets, transport networks serve as an enabling asset, connecting households to diverse food options. This is because the connectivity of an urban area to its peri-urban ecosystem and transportation routes are urban-level variables shaping household food access patterns (81). From a food policy perspective, this result suggests that infrastructure and mobility constraints should be considered part of the nutrition access problem rather than treated as separate development issues for food value chains.

The findings highlight the criticality of infrastructure and utilities, particularly water and energy, in shaping dietary diversity in urban areas. This implies that the functionality of basic household infrastructure strongly influences improved dietary outcomes, likely through better food preparation, storage, and general living conditions. Foods that need a lot of water to produce and prepare, such as fish, fruits and vegetables, are more likely to be found in households with better access to water (82–84). Water and electricity are welfare-enhancing services vital to influence food preparation, food storage, hygiene, and the ability to consume more perishable and nutritionally diverse foods. Reliable energy allows households to conveniently refrigerate perishable, nutrient-dense foods such as meats, fish, dairy, and fresh produce, and to use convenient electric cooking appliances. This exerts immediate impacts on food availability and utilisation from income-mediated effects (85). Households without reliable access to these services may be compelled to rely on simpler, less varied, and less storage-sensitive diets. In Zimbabwe, many urban areas suffer from inadequate infrastructure, including roads, electricity, and water, which hinders the food supply chain and limits the ability of households to store and prepare food. In this sense, the result highlights the importance of understanding dietary diversity through a food systems lens, as nutrition outcomes are a function of interactions between households and the material conditions of the urban service-delivery environment.

In contrast, households obtaining vegetables primarily from formal and informal markets had significantly lower odds of achieving higher dietary diversity than those relying on own production. This implies that relying on markets alone to source vegetables may not yield the required diversified diets, probably because of affordability constraints, weak purchasing power among low-income households, and limited access to a variety of vegetables. One plausible explanation is that households producing vegetables for their own consumption may enjoy more regular physical access to this food group without facing the same cash constraints compared to households that must rely on markets for their supply. The reliance on markets for vegetables may reflect broader exposure to price volatility and affordability barriers, but (86) nevertheless, in urban areas, most poor households are net food consumers. In most urban and peri-urban households in Zimbabwe, vegetables are grown year-round in backyards and consumed daily. Home gardening tends to increase the availability, access, and intake of leafy vegetables compared to other food groups, minimises reliance on intricate market supply chains, and reduces the burden of price volatility and external disruptions (87, 88). However, Olabisi et al. (89) offer a different perspective, arguing that households producing a larger share of their own food attain lower dietary diversity scores than households relying on markets, because their own production limits the set of food items they can produce relative to demand. Thus, to improve household dietary diversity, it may be essential to substantially increase agricultural production diversity to effectuate an impact on dietary diversity (90). The food policy implication is that nutrition-sensitive urban strategies should place greater emphasis on affordability, household production, and the role of informal and peri-urban food sources in sustaining dietary diversity.

Household frequency of meals and dietary diversity are closely related dimensions of household food access and more diversified food consumption patterns. This is similar to a finding in Ethiopia that households with improved daily meal intake were more likely to be classified into a higher dietary diversity cohort, thereby decreasing the probability of falling into a low dietary diversity class (91). This highlights that households with the ability to sustain regular normal meal frequency may also be better positioned to consume a wider variety of food groups, which serves as a key indicator of improved socioeconomic status and micronutrient adequacy. This aligns with related dietary diversity studies, where enhanced frequency of meals has a bearing on improved household dietary outcomes (92–94). However, in many low-income households in urban areas, such as Bindura, the normal frequency of meals does not translate to the required dietary diversity. In Zimbabwe, over a quarter of the country’s urban households resort to skipping meals almost daily, which signals low nutritional intake, and an estimated 54% consume an acceptable diet (95). This is possibly owing to prevailing economic burdens. Thus, awareness is needed to promote frequent intake of different food groups at intervals to maintain adequate dietary intake.

The results of this study highlight the diverse coping strategies households employ in response to consumption shocks and how these affect dietary outcomes. While many food security studies have grounded their investigations in common food-based coping strategies, this study probes the influence of asset- and financial-based coping strategies on household dietary diversity. Urban households relying on asset- and stress-coping strategies such as depleting resources and reducing expenditure indicate deeper vulnerability and often experience lower diversity outcomes. This buttresses the finding that many poor households in Zimbabwe find it difficult to accumulate cash savings, livestock, assets or store food sufficient to protect them from consumption shocks (42). The type of coping strategy adopted by a household signals the level of food and nutritional vulnerability, and more erosive coping responses, such as selling livestock and reducing spending, are associated with poorer dietary diversity outcomes. Asset and stress coping, such as selling livestock, disposing of household assets, or decreasing essential expenditure to cope with income loss (96), often reflects deeper livelihood stress and reduced resilience. In contrast, financial coping strategies such as (97) drawing on savings or borrowing from social networks, may allow households to smooth consumption without immediately undermining their productive base. From a policy lens, this finding supports the need for social protection and livelihood support mechanisms that build resilience capacities to prevent households from resorting to erosive coping responses that may compromise dietary outcomes over time.

### Limitations of the study

This study, despite critical insights, has the following limitations. First, the analysis is based on cross-sectional data and therefore identifies associations rather than causal effects, as some variables, such as the number of meals and coping strategies, may also be jointly determined with dietary diversity and should not be read as exogenous predictors. Second, the sample size used in this study is not representative of all the secondary cities in Zimbabwe. Hence, the results may not be comparable across cities with different socio-economic structures. Third, the cross-sectional design did not account for seasonal variations in food consumption, which longitudinal studies and panel data could address by collecting data across different seasons to better understand temporal trends and their impact on food consumption patterns in urban and peri-urban households. Fourth, while the 24-h dietary recall is widely validated and provides a snapshot of current intake, it may not reflect the person’s usual diet or long-term trends (33). This is limited by the short time window during which households’ dietary diversity can be observed. Alternatively, a structured 7-day recall could determine a household’s consumption pattern. Furthermore, consumption data were collected at the household level rather than individual caloric intake, thus limiting the direct connection between personal-level nutrition and the study. The study does not account for food consumed by household members and instead relies on qualitative measures of HDD. Although the survey data were collected in October 2014 as part of the lead author’s earlier thesis work, the present paper offers a new analysis of those data to examine the structural determinants of household dietary diversity in Bindura. Accordingly, the study results are most appropriately interpreted as historically and analytically informative rather than as a direct reflection of current circumstances in 2026.

### Areas for future research

Building on the findings of this study, several areas warrant further investigation. Future studies should conduct similar research with a larger number of participants in various towns and other secondary cities, including informal settlements, to complement our preliminary findings. Moreover, research using panel data, clearer measures of exposure to the urban food environment, and more explicit modelling of household market access and vulnerability. There is a need for more research on the relationships between cultural nuances and household dietary diversity outcomes in an urban context. There is a need for further research on the measurement and metrics of foods rich in macronutrients. This is because most studies *predictably* use metrics that rely on sums and frequencies of food intake but overlook the quantities of the foods consumed to explore whether they are commensurate with dietary needs (16). For example, an increased frequency of consumption of protein-rich foods does not mean that the required quantity of the macronutrient has increased.

## Conclusions and policy recommendations

This study analysed how socio-economic conditions shape household dietary diversity in Bindura, a secondary city in Zimbabwe, using a historical re-analysis of cross-sectional data from urban and peri-urban households. The *results* showed that in an urban food environment, household dietary diversity was strongly associated with household capability, physical access, infrastructure, food sourcing, consumption pattern and coping behaviour. This indicates that dietary diversity in secondary cities is not only a function of household income or food availability, but of the wider material and access environments through which different households acquire, construct, and sustain diversified diets. The study, therefore, contributes to knowledge of food policy and economics by demonstrating that the quality of diets in secondary African cities is ingrained in a resource-capability matrix, urban service delivery, food-sourcing systems, consumption patterns, and household resilience mechanisms under economic stress. This has important implications for urban food and nutrition policy in Zimbabwe. From a policy perspective, improving dietary diversity in Bindura and comparable urban environments will require more than nutrition messaging alone. It will require integrated interventions that strengthen household access to basic services, improve physical access to food markets by addressing transport constraints, support affordable and reliable food sourcing, and reduce the vulnerability that drives households to rely on erosive coping strategies that undermine diet quality.

## Data Availability

The original contributions presented in the study are included in the article/supplementary material, further inquiries can be directed to the corresponding author.
